# Isolated Myeloid Sarcoma: A Diagnostic Dilemma

**DOI:** 10.7759/cureus.21200

**Published:** 2022-01-13

**Authors:** Hafiz M Aslam, Sindhusha Veeraballi, Zabila Saeed, Andrew Weil, Vijay Chaudhary

**Affiliations:** 1 Hematology and Medical Oncology, East Carolina University, Greenville, USA; 2 Internal Medicine, Saint Michael’s Medical Center, Newark, USA; 3 Internal Medicine/Hematology and Medical Oncology, East Carolina University, Greenville, USA

**Keywords:** cytarabine, aml, testicular mass, leptomeninges, acute myeloid sarcoma

## Abstract

Myeloid sarcoma (MS)/granulocytic sarcoma/myeloblastoma/chloroma is a rare extramedullary proliferation of blast cells of one or more myeloid lineages along with the destruction of the normal architecture of adjacent tissue. Isolated MS is a rare entity with an incidence of 0.7 out of 1 million children and 2 out of 1 million adults. Varied clinical presentation, the rarity of the diagnosis, inadequate immunophenotyping, and lack of available literature makes the disease difficult to manage. Here, we report a case of MS in a 44-year-old male with an initial presentation of testicular mass without bone marrow involvement, causing diagnostic challenges. In this case report, we discuss the pathogenesis, diagnostic challenges, and therapeutic options of MS.

## Introduction

Myeloid sarcoma (MS)/granulocytic sarcoma/myeloblastoma/chloroma is a rare extramedullary proliferation of blast cells of one or more myeloid lineages with the destruction of the normal architecture of adjacent tissue [1,2]. It was first described in 1811. The European Association for Hematopathology has classified extramedullary manifestations of myeloid neoplasms into various subgroups based on clinical history, morphology, immunophenotype, and genetic features. The first subgroup is isolated MS without previous or concurrent myeloid neoplasm, which is a rare entity with an incidence of two cases per million adults. The second is concurrent MS and acute myeloid leukemia (AML) with a focus on karyotypic or molecular findings. The third is MS and AML without recurrent genetic abnormalities. The fourth subgroup is MS presenting as a form of blast transformation in a patient with myelodysplastic neoplasm and/or myeloproliferative neoplasm [1-3]. MS has a slight male predominance, with a male-to-female ratio of 1.2:1. It may occur at any age and on any site of the body, with varied clinical presentations. The most commonly involved anatomical sites are lymph nodes, skin and soft tissues, bone, testes, gastrointestinal tract, and peritoneum [2].

MS is associated with a wide variety of cytogenetic abnormalities. There is limited data available on the prognosis of patients with MS showing a five-year survival rate of 20-30% [3]. A study conducted by Tsimberidou et al. on 20 isolated MS patients revealed that the worst outcome is associated with chromosome 8 abnormalities [4]. The presence of Leukemia cutis (LC) is also considered a marker of aggressive disease with shortened survival and frequent relapses [3,5]. However, statistically significant evidence of worse prognosis with LC was not noted in a study conducted on 381 AML patients [3]. Isolated MS has the potential of progression to AML at a median duration of 5-12 months when the treatment is delayed or inadequate [3]. Here, we report the case of a 44-year-old male with extramedullary myeloid neoplasm involving the testis, skin, and leptomeninges with diagnostic and therapeutic challenges. This case is rare is due to the initial presentation of MS without bone marrow involvement.

## Case presentation

**Figure 1:**
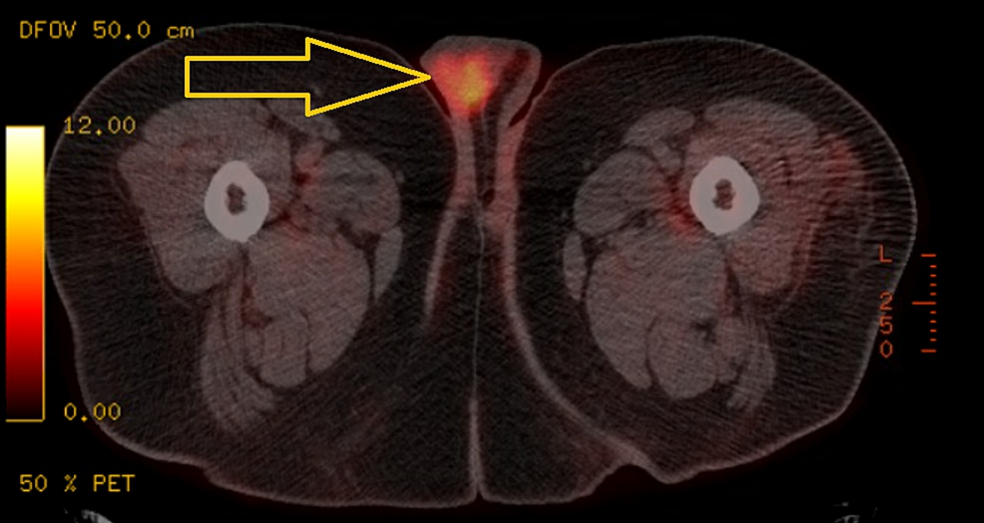
PET-CT, status post-bilateral orchiectomy, revealing an 8.0 SUV focus of hypermetabolic activity in the right hemiscrotum concerning for locally recurrent disease. PET-CT: positron emission tomography-computed tomography; SUV: standardized uptake value

A 44-year-old male with a medical history of hypertension, diabetes mellitus, and morbid obesity presented with right testicular pain and swelling. He underwent radical orchiectomy. Pathology results showed classical seminoma initially with surgical margins and lymphovascular invasion, for which he received adjuvant carboplatin for pT3 disease. He developed left testicular pain and swelling two months later and underwent left radical orchiectomy. Pathology reported CD4+, CD56+, high-grade hematopoietic neoplasm. It was sent for a second opinion to the National Institutes of Health and was consistent with MS with monoblastic features (intermediate risk for AML, normal next-generation sequencing, MDS fluorescence in situ hybridization and cytogenetics). Repeat evaluation of right testicular specimen was also CD4+. Bone marrow biopsy showed normocellular marrow with multilineage hematopoiesis. A positron emission tomography (PET) scan revealed an 8.0 standardized uptake value focus of hypermetabolic activity in the right hemiscrotum (Figure 1).

**Figure 2:**
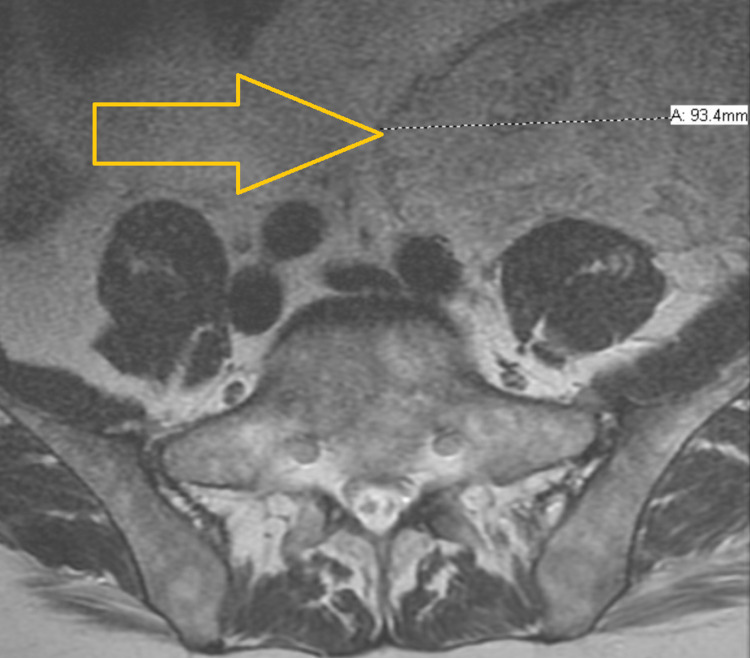
MRI of the spine showing a very large, heterogeneously enhancing, mixed-signal, mass-like lesion in the left hemipelvis. MRI: magnetic resonance imaging

PET scan also showed widespread osseous areas of increased uptake and three soft tissue nodules within the subcutaneous tissues of the left abdominal wall. Fine needle aspiration of a subcutaneous nodule showed CD56+ monocytoid cells. Induction chemotherapy with 7+3 (cytarabine, daunorubicin) and gemtuzumab on days one, four, and seven was completed. Lumbar puncture studies showed monoblastic/monocytic proliferation, and the patient received intrathecal (IT) chemotherapy alternating between methotrexate and cytarabine every week. Cerebrospinal fluid (CSF) findings cleared after two IT chemotherapy treatments. The patient remained in the hospital for 87 days due to poor count recovery and development of pulmonary embolism. A repeat PET scan showed seven areas of hypermetabolic foci involving nodular densities of the bilateral lower anterior abdominal wall. Biopsy of one of these lesions showed fat necrosis. He completed two cycles of high-dose cytarabine but had repeated hospital admissions due to chemotherapy and MS complication. Hence, his therapy was switched to azacytidine and venetoclax. Meanwhile, the patient was referred for a bone marrow transplant. He had disease progression after eight months of total therapy when he presented with back pain and lower extremity weakness. Magnetic resonance imaging of the brain and spine showed a new, patchy T2 fluid-attenuated inversion recovery hyperintense signal in the right frontal white matter and increased number and size of marrow replacing lesions throughout the visualized skeleton (Figure 2).

The patient underwent a bone biopsy that showed >90% marrow involvement of previously diagnosed MS (sheets of infiltrative cells with identical phenotype. He was positive for CD56 (>90% of marrow cellularity), CD4, and lysozyme but negative for synaptophysin, chromogranin, S100, CD117, CD34, CD20, CD3, C8, MPO, and CD138. His hospital course was complicated by acute kidney injury, electrolyte imbalance, and hemodynamically instability requiring vasopressor support. Discussions were held for re-induction with CLAG (cladribine 5 mg/m^2^ and cytarabine 2 g/m^2^) versus best supportive care. The patient made the decision to pursue comfort care and was transferred to the palliative care floor where he passed away peacefully.

## Discussion

Isolated MS is the extramedullary proliferation of immature myeloid cells without the involvement of bone marrow. The incidence of isolated MS is approximately 0.7 out of 1 million children and 2 out of 1 million adults. Central nervous system (CNS) involvement of MS is extremely rare. In a large population-based cohort study conducted in Denmark, only 0.4% of patients were found to have CNS involvement [6]. Cutaneous granulocytic sarcoma/LC is an infiltration of neoplastic leukocytes involving the epidermis, dermis, and subcutis [2,5]. The occurrence of LC without bone marrow involvement is termed “aleukemic leukemia cutis,” and has a high risk of progression to AML. LC is associated with poor outcomes, a high recurrence rate, more aggressive disease, and shortened survival. The lower extremities followed by the upper extremities, back, trunk, and face are the most commonly involved anatomical sites. In our patient, the anterior abdominal wall was involved [2].

The pathogenesis of MS is unclear and can be attributed to an aberrant homing signal expressed on tissue, chemokine receptors/ligands, and RAS-mitogen-activated protein kinase/extracellular signal-regulated kinase signaling facilitating the migration of AML subclones [4]. AML subclones have been shown to express high levels of enhancer of Zeste 2 (EZH2) and are correlated with extramedullary infiltration of AML. Moreover, the interactions between matrix metalloproteinase (MMP)-9 and leukocyte beta 2 integrin along with unidentified proteins facilitate the migration of AML cells into the non-myeloid region. EZH2 causes upregulation of tissue inhibitor of metalloproteinases leading to overexpression of MMPs. The uninhibited MMPs facilitate the escape of blast cells in the extramedullary space by degrading the extracellular matrix [1]. Cytogenetic abnormalities such as t(8;21), t(16;16), (q22;q22), inv(16), mixed lineage leukemia rearrangements, trisomies of 4, 8, 11, monosomy 7, deletions of chromosomes 5q, 16q, and 20q, FMS-like tyrosine kinase 3-internal tandem duplication, and nucleophosmin-1 mutation are frequently detected in MS patients. However, the role of cytogenetic abnormalities in the pathophysiology is unknown [2,7]. Immunohistochemistry of MS is frequently positive for myeloperoxidase expressed in 66-96% cases. In addition, CD43, CD34, CD45, CD56, CD68, lysozyme, CD117, CD11c, CD13, CD33, and B and T cell markers can also be observed [3,8].

Diagnostic and therapeutic challenges of MS can be attributed to the varied clinical presentation, the rarity of the case, inadequate immunophenotypic features, and lack of available literature. MS should always be considered in the differential diagnosis of a small round blue cell tumor and should be more seriously suspected if eosinophilic myelocytes are seen on hematoxylin and eosin-stained biopsies. There is a greater tendency to misdiagnose MS as malignant lymphoma, poorly differentiated carcinoma, or other hematological neoplasms due to radiological and histologic similarities [8]. A previous retrospective series reported the rate of misdiagnosis to be 75%. However, the most recent series observed that the rate of misdiagnosis was 25-47%, which is significant [8]. Hence, a comprehensive analysis of clinical and imaging features, tissue biopsy, immunohistochemistry, and molecular analysis is necessary. Diagnosis mostly relies on a high index of suspicion.

Due to the rarity of the case and lack of randomized clinical trials, there is no standardized treatment strategy. The modalities of treatment available include systemic chemotherapy, local radiotherapy, allogeneic bone marrow transplantation, targeted therapy, and immunotherapy [2,9]. Local therapy with surgery or radiotheraphy alone is considered ineffective due to the high risk of recurrence and progression to AML. However, local therapy combined with systemic chemotherapy is a preferred option. Various AML chemotherapy-like regimens have been used in MS, including idarubicin and cytarabine; cyclophosphamide, cytarabine, topotecan, and granulocyte colony-stimulating factor (G-CSF) (CATG); fludarabine, cytarabine, idarubicin, and G-CSF (FLAG); and daunorubicin and cytarabine [9]. Tsimberidou et al. reported a 69% complete remission rate of isolated MS with cytarabine, idarubicin, and fludarabine [4]. Cytarabine-based chemotherapy regimens are most commonly used in MS [7]. In our patient, cytarabine and daunorubicin with gemtuzumab were started initially and later switched to azacytidine and venetoclax. Favorable outcomes with allogeneic or autologous hematopoietic cell transplantation have been reported in several retrospective studies. Five-year survival rates of 48% have been reported with allogeneic stem cell transplantation (alloSCT) [2,9]. Another study reported an overall survival rate of 76% with auto/alloSCT at 48 months compared to 0% in non-transplant patients [7]. Further studies are needed to determine the role of SCT as first-line treatment in MS after induction of remission [9]. A humanized anti-CD33 monoclonal antibody, tyrosine kinase inhibitors, immune checkpoint inhibitors like anti-cytotoxic T-lymphocyte-associated protein 4 (ipilimumab) have been used in MS. However, complete understanding of the genetic profile is necessary to investigate the role of novel targeted therapies and develop prospective multicenter controlled trials [9-12].

## Conclusions

There are several challenges in the diagnosis of isolated MS, causing delay in the appropriate treatment. Diagnosis of MS mostly relies on a high index of suspicion. Hence, MS should be considered as a differential diagnosis for any atypical cellular infiltrate. Increased awareness is needed among physicians regarding MS to attenuate the rate of misdiagnosis. A staged diagnostic approach with history, clinical suspicion, imaging, tissue biopsy, immunohistochemistry, and molecular analysis is crucial to make the correct diagnosis. Systemic chemotherapy using AML-like regimens should be commenced early, even in non-leukemic disease. AlloSCT should be considered in most patients fit enough to undergo transplants. Further understanding of the genetic profile and pathogenesis is needed for more refined diagnostic and therapeutic decisions.
